# Supplementary material for: Increased fronto-temporal connectivity by modified melody in real music
Source: PLoS One. 2020 Jul 8;15(7):e0235770. doi: 10.1371/journal.pone.0235770 (PMC7343137; doi:10.1371/journal.pone.0235770)
Supplement: S1 Table — (DOCX) [file pone.0235770.s001.docx]

**S1 Table. Mean and SD values for LTDMIs from the left HG to the right HG, the left IFG, and the right IFG.**

|  | ***lHG* → *rHG*** | | | | ***lHG* → *lIFG*** | | | | ***lHG* → *rIFG*** | | | |
| --- | --- | --- | --- | --- | --- | --- | --- | --- | --- | --- | --- | --- |
|  | ***V1*** | ***V2*** | ***V3*** | ***V4*** | ***V1*** | ***V2*** | ***V3*** | ***V4*** | ***V1*** | ***V2*** | ***V3*** | ***V4*** |
| ***S01*** | 0.0163 | 0.0142 | 0.0454 | 0.0191 | 0.0884 | 0.1131 | 0.0962 | 0.0734 | 0.0119 | 0.0197 | 0.0435 | 0.0036 |
| ***S02*** | 0.0342 | 0.0464 | 0.0299 | 0.0124 | 0.0376 | 0.0651 | 0.0567 | 0.0349 | 0.0066 | 0.0153 | 0.0112 | 0.0208 |
| ***S03*** | 0.0261 | 0.0389 | 0.0170 | 0.0689 | 0.0341 | 0.0420 | 0.2038 | 0.0775 | 0.0230 | 0.0667 | 0.0520 | 0.1063 |
| ***S04*** | 0.0101 | 0.0085 | 0.0168 | 0.0022 | 0.0694 | 0.0827 | 0.0456 | 0.0689 | 0.0047 | 0.0011 | 0.0243 | 0.0075 |
| ***S05*** | 0.0403 | 0.0264 | 0.0075 | 0.0109 | 0.0576 | 0.1010 | 0.0345 | 0.0331 | 0.0018 | 0.0342 | 0.0247 | 0.0084 |
| ***S06*** | 0.0230 | 0.0222 | 0.0027 | 0.0253 | 0.1415 | 0.0583 | 0.0236 | 0.0357 | 0.0062 | 0.0034 | 0.0143 | 0.0146 |
| ***S07*** | 0.0112 | 0.0080 | 0.0349 | 0.0222 | 0.1054 | 0.0589 | 0.0852 | 0.0638 | 0.0353 | 0.0332 | 0.0429 | 0.0069 |
| ***S08*** | 0.0238 | 0.0162 | 0.0312 | 0.0067 | 0.0660 | 0.0051 | 0.0899 | 0.0455 | 0.0110 | 0.0335 | 0.0253 | 0.0188 |
| ***S09*** | 0.0090 | 0.0047 | 0.0587 | 0.0377 | 0.0017 | 0.0726 | 0.0308 | 0.0177 | 0.0754 | 0.0247 | 0.0228 | 0.0049 |
| ***S10*** | 0.0039 | 0.0041 | 0.0146 | 0.0107 | 0.0797 | 0.0451 | 0.0790 | 0.0491 | 0.0087 | 0.0061 | 0.0443 | 0.0079 |
| ***S11*** | 0.0055 | 0.0126 | 0.0086 | 0.0266 | 0.1140 | 0.1056 | 0.0346 | 0.0881 | 0.0163 | 0.0077 | 0.0078 | 0.0285 |
| ***S12*** | 0.0022 | 0.0497 | 0.0653 | 0.0175 | 0.0567 | 0.0072 | 0.0282 | 0.0317 | 0.0415 | 0.0416 | 0.0042 | 0.0097 |
| ***S13*** | 0.0446 | 0.0180 | 0.0359 | 0.0009 | 0.0552 | 0.0524 | 0.1112 | 0.1002 | 0.0070 | 0.0365 | 0.0312 | 0.0295 |
| ***S14*** | 0.0091 | 0.1005 | 0.0120 | 0.0287 | 0.0050 | 0.0256 | 0.0057 | 0.0529 | 0.0100 | 0.0611 | 0.0041 | 0.0164 |
| ***S15*** | 0.0270 | 0.0029 | 0.0312 | 0.0135 | 0.0470 | 0.0048 | 0.0606 | 0.0285 | 0.0101 | 0.0029 | 0.0025 | 0.0148 |
| ***S16*** | 0.0525 | 0.0294 | 0.0049 | 0.0072 | 0.1698 | 0.0449 | 0.1113 | 0.0839 | 0.0436 | 0.0089 | 0.0014 | 0.0128 |
| ***S17*** | 0.0320 | 0.0446 | 0.0245 | 0.0306 | 0.0107 | 0.0247 | 0.0379 | 0.0017 | 0.0410 | 0.0232 | 0.0611 | 0.0081 |
| ***S18*** | 0.0020 | 0.0165 | 0.0107 | 0.0191 | 0.0894 | 0.0040 | 0.0191 | 0.0244 | 0.0076 | 0.0245 | 0.0072 | 0.0385 |
| ***S19*** | 0.0310 | 0.0192 | 0.0416 | 0.0157 | 0.0695 | 0.0325 | 0.0617 | 0.0322 | 0.0203 | 0.0106 | 0.0170 | 0.0043 |
| ***S20*** | 0.0209 | 0.0061 | 0.0192 | 0.0407 | 0.0109 | 0.0987 | 0.0407 | 0.0021 | 0.0087 | 0.0099 | 0.0029 | 0.0080 |
| ***S21*** | 0.0058 | 0.0026 | 0.0256 | 0.0180 | 0.0156 | 0.0023 | 0.0022 | 0.0129 | 0.0039 | 0.0023 | 0.0065 | 0.0228 |
| ***S22*** | 0.0017 | 0.0038 | 0.0030 | 0.0179 | 0.1559 | 0.1445 | 0.0533 | 0.1955 | 0.0342 | 0.0064 | 0.0027 | 0.0122 |
| ***S23*** | 0.0121 | 0.0166 | 0.0543 | 0.0325 | 0.0903 | 0.0801 | 0.0757 | 0.0310 | 0.0218 | 0.0100 | 0.0185 | 0.0134 |
| ***S24*** | 0.0194 | 0.0136 | 0.0137 | 0.0084 | 0.0782 | 0.0819 | 0.1100 | 0.0756 | 0.0144 | 0.0207 | 0.0096 | 0.0169 |
| ***S25*** | 0.0157 | 0.0073 | 0.0266 | 0.0383 | 0.0311 | 0.1052 | 0.1716 | 0.0556 | 0.0106 | 0.0187 | 0.0140 | 0.0115 |
| ***MEAN*** | 0.0192 | 0.0213 | 0.0254 | 0.0213 | 0.0672 | 0.0583 | 0.0668 | 0.0526 | 0.0190 | 0.0209 | 0.0198 | 0.0179 |
| ***SD*** | 0.0141 | 0.0217 | 0.0175 | 0.0149 | 0.0459 | 0.0398 | 0.0486 | 0.0401 | 0.0173 | 0.0176 | 0.0172 | 0.0203 |

*Abbreviations*: lSTG = left STG, rSTG = right STG, lIFG = left IFG, rIFG = right IFG, V1 = Variation I, V2 = Variation II,

V3 = Variation III, V4 = Variation IV.
