# Supplementary material for: Increased fronto-temporal connectivity by modified melody in real music
Source: PLoS One. 2020 Jul 8;15(7):e0235770. doi: 10.1371/journal.pone.0235770 (PMC7343137; doi:10.1371/journal.pone.0235770)
Supplement: S2 Table — (DOCX) [file pone.0235770.s002.docx]

**S2 Table. Mean and SD values for LTDMIs from the right HG to the left HG, the left IFG, and the right IFG.**

|  | ***rHG* → *lHG*** | | | | ***rHG* → *lIFG*** | | | | ***rHG* → *rIFG*** | | | |
| --- | --- | --- | --- | --- | --- | --- | --- | --- | --- | --- | --- | --- |
|  | ***V1*** | ***V2*** | ***V3*** | ***V4*** | ***V1*** | ***V2*** | ***V3*** | ***V4*** | ***V1*** | ***V2*** | ***V3*** | ***V4*** |
| ***S01*** | 0.0279 | 0.0284 | 0.0218 | 0.0369 | 0.0118 | 0.0024 | 0.0164 | 0.0262 | 0.1147 | 0.0364 | 0.1071 | 0.0404 |
| ***S02*** | 0.0199 | 0.0102 | 0.0041 | 0.0278 | 0.0757 | 0.0230 | 0.0090 | 0.0018 | 0.0236 | 0.0125 | 0.0193 | 0.0363 |
| ***S03*** | 0.0042 | 0.0135 | 0.0052 | 0.0651 | 0.0031 | 0.0153 | 0.0008 | 0.0122 | 0.0785 | 0.1730 | 0.0817 | 0.1335 |
| ***S04*** | 0.0245 | 0.0075 | 0.0309 | 0.0174 | 0.0466 | 0.0070 | 0.0304 | 0.0115 | 0.0814 | 0.1159 | 0.0628 | 0.1742 |
| ***S05*** | 0.0281 | 0.0279 | 0.0239 | 0.0331 | 0.0146 | 0.0121 | 0.0098 | 0.0206 | 0.0154 | 0.0318 | 0.0670 | 0.0548 |
| ***S06*** | 0.0280 | 0.0228 | 0.0130 | 0.0413 | 0.0155 | 0.0117 | 0.0073 | 0.0055 | 0.0378 | 0.0181 | 0.0901 | 0.0546 |
| ***S07*** | 0.0163 | 0.0119 | 0.0358 | 0.0033 | 0.0375 | 0.0038 | 0.0482 | 0.0069 | 0.0762 | 0.0527 | 0.0309 | 0.0129 |
| ***S08*** | 0.0053 | 0.0115 | 0.0150 | 0.0143 | 0.0108 | 0.0258 | 0.0077 | 0.0016 | 0.0510 | 0.0168 | 0.0680 | 0.0216 |
| ***S09*** | 0.0297 | 0.0014 | 0.0454 | 0.0425 | 0.0066 | 0.0039 | 0.0121 | 0.0309 | 0.0585 | 0.0664 | 0.0544 | 0.0096 |
| ***S10*** | 0.0029 | 0.0046 | 0.0117 | 0.0097 | 0.0130 | 0.0020 | 0.0180 | 0.0025 | 0.0650 | 0.0553 | 0.1369 | 0.0810 |
| ***S11*** | 0.0060 | 0.0040 | 0.0159 | 0.0309 | 0.0167 | 0.0046 | 0.0026 | 0.0202 | 0.0578 | 0.1540 | 0.0456 | 0.1703 |
| ***S12*** | 0.0033 | 0.0193 | 0.0261 | 0.0386 | 0.0115 | 0.0078 | 0.0245 | 0.0199 | 0.0062 | 0.1197 | 0.0136 | 0.0024 |
| ***S13*** | 0.0315 | 0.0316 | 0.0413 | 0.0170 | 0.0526 | 0.0586 | 0.0724 | 0.0282 | 0.0949 | 0.0720 | 0.0965 | 0.0728 |
| ***S14*** | 0.0267 | 0.0337 | 0.0169 | 0.0112 | 0.0048 | 0.0048 | 0.0355 | 0.0138 | 0.0747 | 0.0778 | 0.0670 | 0.0927 |
| ***S15*** | 0.0114 | 0.0056 | 0.0518 | 0.0119 | 0.0124 | 0.0171 | 0.0338 | 0.0211 | 0.0143 | 0.0068 | 0.0388 | 0.0575 |
| ***S16*** | 0.0683 | 0.0440 | 0.0006 | 0.0033 | 0.0661 | 0.0104 | 0.0080 | 0.0234 | 0.1290 | 0.1275 | 0.1589 | 0.1271 |
| ***S17*** | 0.0627 | 0.0515 | 0.0226 | 0.0097 | 0.0393 | 0.0448 | 0.0087 | 0.0102 | 0.0168 | 0.0494 | 0.0065 | 0.0080 |
| ***S18*** | 0.0226 | 0.0260 | 0.0412 | 0.0267 | 0.0115 | 0.0092 | 0.0144 | 0.0389 | 0.0030 | 0.0557 | 0.0242 | 0.0887 |
| ***S19*** | 0.0154 | 0.0182 | 0.0200 | 0.0052 | 0.0094 | 0.0113 | 0.0183 | 0.0126 | 0.0125 | 0.0395 | 0.0498 | 0.0142 |
| ***S20*** | 0.0269 | 0.0013 | 0.0107 | 0.0049 | 0.0134 | 0.0020 | 0.0035 | 0.0035 | 0.0030 | 0.0660 | 0.0225 | 0.0035 |
| ***S21*** | 0.0154 | 0.0097 | 0.0075 | 0.0031 | 0.0108 | 0.0195 | 0.0121 | 0.0140 | 0.0373 | 0.0390 | 0.0139 | 0.0708 |
| ***S22*** | 0.0034 | 0.0047 | 0.0083 | 0.0064 | 0.0124 | 0.0022 | 0.0365 | 0.0016 | 0.1008 | 0.1180 | 0.0934 | 0.1351 |
| ***S23*** | 0.0371 | 0.0067 | 0.0160 | 0.0480 | 0.0012 | 0.0007 | 0.0154 | 0.0035 | 0.0257 | 0.0091 | 0.0496 | 0.0568 |
| ***S24*** | 0.0323 | 0.0155 | 0.0131 | 0.0169 | 0.0059 | 0.0075 | 0.0121 | 0.0011 | 0.0161 | 0.0458 | 0.0276 | 0.0464 |
| ***S25*** | 0.0038 | 0.0154 | 0.0036 | 0.0205 | 0.0089 | 0.0433 | 0.0017 | 0.0104 | 0.0130 | 0.0060 | 0.0077 | 0.0176 |
| ***MEAN*** | 0.0221 | 0.0171 | 0.0201 | 0.0218 | 0.0205 | 0.0140 | 0.0184 | 0.0137 | 0.0483 | 0.0626 | 0.0574 | 0.0633 |
| ***SD*** | 0.0170 | 0.0133 | 0.0140 | 0.0166 | 0.0202 | 0.0149 | 0.0166 | 0.0105 | 0.0375 | 0.0472 | 0.0402 | 0.0515 |

*Abbreviations*: lSTG = left STG, rSTG = right STG, lIFG = left IFG, rIFG = right IFG, V1 = Variation I, V2 = Variation II,

V3 = Variation III, V4 = Variation IV.
