# Supplementary material for: Increased fronto-temporal connectivity by modified melody in real music
Source: PLoS One. 2020 Jul 8;15(7):e0235770. doi: 10.1371/journal.pone.0235770 (PMC7343137; doi:10.1371/journal.pone.0235770)
Supplement: S3 Table — (DOCX) [file pone.0235770.s003.docx]

**S3 Table. Mean and SD values for LTDMIs from the left IFG to the left HG, the right HG, and the right IFG.**

|  | ***lIFG* → *lHG*** | | | | ***lIFG* → *rHG*** | | | | ***lIFG* → *rIFG*** | | | |
| --- | --- | --- | --- | --- | --- | --- | --- | --- | --- | --- | --- | --- |
|  | ***V1*** | ***V2*** | ***V3*** | ***V4*** | ***V1*** | ***V2*** | ***V3*** | ***V4*** | ***V1*** | ***V2*** | ***V3*** | ***V4*** |
| ***S01*** | 0.0464 | 0.0688 | 0.0735 | 0.0870 | 0.0129 | 0.0057 | 0.0619 | 0.0103 | 0.0042 | 0.0151 | 0.0431 | 0.0054 |
| ***S02*** | 0.0435 | 0.0303 | 0.0581 | 0.0272 | 0.1071 | 0.0198 | 0.0101 | 0.0142 | 0.0191 | 0.0073 | 0.0152 | 0.0356 |
| ***S03*** | 0.0417 | 0.0340 | 0.1837 | 0.0693 | 0.0023 | 0.0120 | 0.0150 | 0.0076 | 0.0058 | 0.0142 | 0.0440 | 0.0257 |
| ***S04*** | 0.0674 | 0.0801 | 0.0599 | 0.1003 | 0.0215 | 0.0114 | 0.0117 | 0.0107 | 0.0155 | 0.0154 | 0.0327 | 0.0022 |
| ***S05*** | 0.0984 | 0.0925 | 0.0479 | 0.0171 | 0.0144 | 0.0108 | 0.0079 | 0.0046 | 0.0053 | 0.0216 | 0.0057 | 0.0341 |
| ***S06*** | 0.0817 | 0.0440 | 0.0139 | 0.0604 | 0.0135 | 0.0024 | 0.0204 | 0.0028 | 0.0034 | 0.0959 | 0.0063 | 0.0099 |
| ***S07*** | 0.0715 | 0.0420 | 0.0849 | 0.0677 | 0.0136 | 0.0085 | 0.0486 | 0.0066 | 0.0223 | 0.0152 | 0.0383 | 0.0028 |
| ***S08*** | 0.0837 | 0.0243 | 0.0625 | 0.0330 | 0.0353 | 0.0018 | 0.0360 | 0.0192 | 0.0100 | 0.0034 | 0.0138 | 0.0297 |
| ***S09*** | 0.0306 | 0.0949 | 0.0555 | 0.0105 | 0.0068 | 0.0039 | 0.0211 | 0.0114 | 0.0210 | 0.0290 | 0.0042 | 0.0220 |
| ***S10*** | 0.0768 | 0.0970 | 0.0402 | 0.0484 | 0.0138 | 0.0027 | 0.0028 | 0.0067 | 0.0208 | 0.0096 | 0.0236 | 0.0240 |
| ***S11*** | 0.1692 | 0.1120 | 0.0668 | 0.0849 | 0.0109 | 0.0080 | 0.0238 | 0.0076 | 0.0389 | 0.0094 | 0.0113 | 0.0576 |
| ***S12*** | 0.0421 | 0.0106 | 0.0248 | 0.0518 | 0.0240 | 0.0021 | 0.0415 | 0.0089 | 0.0419 | 0.0004 | 0.0071 | 0.0147 |
| ***S13*** | 0.0447 | 0.0599 | 0.1195 | 0.0651 | 0.0418 | 0.0474 | 0.0377 | 0.0200 | 0.0463 | 0.0775 | 0.0559 | 0.0365 |
| ***S14*** | 0.0111 | 0.0292 | 0.0199 | 0.1121 | 0.0065 | 0.0066 | 0.0209 | 0.0274 | 0.0107 | 0.0064 | 0.0705 | 0.0794 |
| ***S15*** | 0.0430 | 0.0232 | 0.1173 | 0.0152 | 0.0445 | 0.0105 | 0.0493 | 0.0164 | 0.0144 | 0.0305 | 0.0398 | 0.0564 |
| ***S16*** | 0.1267 | 0.1054 | 0.1596 | 0.0615 | 0.0386 | 0.0275 | 0.0066 | 0.0290 | 0.0230 | 0.0134 | 0.0104 | 0.0145 |
| ***S17*** | 0.0416 | 0.0445 | 0.0432 | 0.0093 | 0.0407 | 0.0231 | 0.0100 | 0.0043 | 0.0344 | 0.0050 | 0.0596 | 0.0106 |
| ***S18*** | 0.0818 | 0.0040 | 0.0080 | 0.0214 | 0.0027 | 0.0074 | 0.0276 | 0.0073 | 0.0280 | 0.0031 | 0.0161 | 0.0189 |
| ***S19*** | 0.1042 | 0.0160 | 0.0417 | 0.0329 | 0.0258 | 0.0157 | 0.0136 | 0.0089 | 0.0167 | 0.0021 | 0.0356 | 0.0055 |
| ***S20*** | 0.0174 | 0.1173 | 0.0425 | 0.0145 | 0.0182 | 0.0011 | 0.0122 | 0.0132 | 0.0097 | 0.0191 | 0.0230 | 0.0215 |
| ***S21*** | 0.0182 | 0.0120 | 0.0024 | 0.0097 | 0.0378 | 0.0169 | 0.0038 | 0.0089 | 0.0259 | 0.0249 | 0.0728 | 0.0358 |
| ***S22*** | 0.0894 | 0.0779 | 0.0442 | 0.1261 | 0.0131 | 0.0037 | 0.0225 | 0.0059 | 0.0113 | 0.0112 | 0.1028 | 0.0030 |
| ***S23*** | 0.0612 | 0.0500 | 0.1254 | 0.0766 | 0.0059 | 0.0071 | 0.0539 | 0.0047 | 0.0263 | 0.0085 | 0.0315 | 0.0092 |
| ***S24*** | 0.0805 | 0.0337 | 0.1133 | 0.0898 | 0.0093 | 0.0010 | 0.0026 | 0.0018 | 0.0086 | 0.0035 | 0.0102 | 0.0022 |
| ***S25*** | 0.0572 | 0.1292 | 0.1552 | 0.0422 | 0.0288 | 0.0269 | 0.0216 | 0.0092 | 0.0103 | 0.0134 | 0.0365 | 0.0289 |
| ***MEAN*** | 0.0652 | 0.0573 | 0.0706 | 0.0534 | 0.0236 | 0.0114 | 0.0233 | 0.0107 | 0.0190 | 0.0182 | 0.0324 | 0.0234 |
| ***SD*** | 0.0360 | 0.0375 | 0.0496 | 0.0343 | 0.0218 | 0.0109 | 0.0171 | 0.0070 | 0.0120 | 0.0223 | 0.0250 | 0.0195 |

*Abbreviations*: lSTG = left STG, rSTG = right STG, lIFG = left IFG, rIFG = right IFG, V1 = Variation I, V2 = Variation II,

V3 = Variation III, V4 = Variation IV.
