# Supplementary material for: Increased fronto-temporal connectivity by modified melody in real music
Source: PLoS One. 2020 Jul 8;15(7):e0235770. doi: 10.1371/journal.pone.0235770 (PMC7343137; doi:10.1371/journal.pone.0235770)
Supplement: S4 Table — (DOCX) [file pone.0235770.s004.docx]

**S4 Table. Mean and SD values for LTDMIs from the right IFG to the left HG, the right HG, and the left IFG.**

|  | ***rIFG* → *lHG*** | | | | ***rIFG* → *rHG*** | | | | ***rIFG* → *lIFG*** | | | |
| --- | --- | --- | --- | --- | --- | --- | --- | --- | --- | --- | --- | --- |
|  | ***V1*** | ***V2*** | ***V3*** | ***V4*** | ***V1*** | ***V2*** | ***V3*** | ***V4*** | ***V1*** | ***V2*** | ***V3*** | ***V4*** |
| ***S01*** | 0.0107 | 0.0239 | 0.0263 | 0.0222 | 0.0862 | 0.0390 | 0.1435 | 0.0855 | 0.0026 | 0.0234 | 0.0107 | 0.0341 |
| ***S02*** | 0.0108 | 0.0077 | 0.0058 | 0.0106 | 0.0704 | 0.0188 | 0.0350 | 0.1093 | 0.0420 | 0.0129 | 0.0134 | 0.0089 |
| ***S03*** | 0.0067 | 0.0358 | 0.0105 | 0.1043 | 0.1058 | 0.1906 | 0.0802 | 0.1687 | 0.0127 | 0.0131 | 0.0299 | 0.0374 |
| ***S04*** | 0.0190 | 0.0120 | 0.0096 | 0.0337 | 0.0936 | 0.1254 | 0.0345 | 0.1028 | 0.0592 | 0.0056 | 0.0285 | 0.0306 |
| ***S05*** | 0.0186 | 0.0110 | 0.0214 | 0.0127 | 0.0188 | 0.0181 | 0.0678 | 0.0322 | 0.0351 | 0.0135 | 0.0042 | 0.0270 |
| ***S06*** | 0.0017 | 0.0017 | 0.0150 | 0.0440 | 0.0303 | 0.0417 | 0.0771 | 0.0859 | 0.0206 | 0.0876 | 0.0025 | 0.0150 |
| ***S07*** | 0.0591 | 0.0135 | 0.0392 | 0.0281 | 0.0402 | 0.0671 | 0.0711 | 0.0017 | 0.0449 | 0.0070 | 0.0521 | 0.0010 |
| ***S08*** | 0.0068 | 0.0312 | 0.0334 | 0.0096 | 0.0198 | 0.0099 | 0.1212 | 0.0160 | 0.0044 | 0.0234 | 0.0191 | 0.0119 |
| ***S09*** | 0.0645 | 0.0300 | 0.0274 | 0.0134 | 0.0213 | 0.0401 | 0.0613 | 0.0188 | 0.0233 | 0.0124 | 0.0037 | 0.0288 |
| ***S10*** | 0.0176 | 0.0189 | 0.0190 | 0.0135 | 0.1241 | 0.0723 | 0.1037 | 0.0914 | 0.0211 | 0.0059 | 0.0269 | 0.0193 |
| ***S11*** | 0.0135 | 0.0056 | 0.0526 | 0.0055 | 0.0770 | 0.1052 | 0.0349 | 0.1467 | 0.0186 | 0.0058 | 0.0347 | 0.0441 |
| ***S12*** | 0.0252 | 0.0325 | 0.0131 | 0.0227 | 0.0029 | 0.1646 | 0.0123 | 0.0103 | 0.0134 | 0.0023 | 0.0024 | 0.0177 |
| ***S13*** | 0.0048 | 0.0161 | 0.0086 | 0.0087 | 0.1156 | 0.0698 | 0.0354 | 0.0514 | 0.0712 | 0.0637 | 0.0621 | 0.0172 |
| ***S14*** | 0.0363 | 0.0426 | 0.0078 | 0.0380 | 0.0178 | 0.0860 | 0.0237 | 0.0913 | 0.0101 | 0.0058 | 0.0350 | 0.0412 |
| ***S15*** | 0.0308 | 0.0008 | 0.0125 | 0.0235 | 0.0123 | 0.0160 | 0.0450 | 0.0557 | 0.0477 | 0.0352 | 0.0252 | 0.0086 |
| ***S16*** | 0.0455 | 0.0214 | 0.0003 | 0.0047 | 0.1422 | 0.1834 | 0.1607 | 0.1301 | 0.0443 | 0.0033 | 0.0064 | 0.0071 |
| ***S17*** | 0.0229 | 0.0078 | 0.0500 | 0.0056 | 0.0320 | 0.0535 | 0.0038 | 0.0008 | 0.0315 | 0.0078 | 0.0294 | 0.0191 |
| ***S18*** | 0.0514 | 0.0180 | 0.0022 | 0.0343 | 0.0019 | 0.0392 | 0.0580 | 0.0724 | 0.0515 | 0.0011 | 0.0134 | 0.0278 |
| ***S19*** | 0.0154 | 0.0055 | 0.0283 | 0.0222 | 0.0349 | 0.0746 | 0.0239 | 0.0249 | 0.0106 | 0.0020 | 0.0451 | 0.0196 |
| ***S20*** | 0.0043 | 0.0074 | 0.0115 | 0.0186 | 0.0094 | 0.0484 | 0.0412 | 0.0069 | 0.0034 | 0.0059 | 0.0062 | 0.0121 |
| ***S21*** | 0.0089 | 0.0196 | 0.0206 | 0.0327 | 0.0391 | 0.0694 | 0.0052 | 0.0273 | 0.0471 | 0.0257 | 0.0525 | 0.0189 |
| ***S22*** | 0.0239 | 0.0037 | 0.0089 | 0.0038 | 0.0774 | 0.1227 | 0.0387 | 0.1649 | 0.0046 | 0.0128 | 0.0469 | 0.0010 |
| ***S23*** | 0.0067 | 0.0150 | 0.0117 | 0.0029 | 0.0302 | 0.0113 | 0.0569 | 0.0684 | 0.0420 | 0.0105 | 0.0273 | 0.0303 |
| ***S24*** | 0.0349 | 0.0126 | 0.0047 | 0.0247 | 0.0196 | 0.0666 | 0.0373 | 0.0932 | 0.0007 | 0.0091 | 0.0178 | 0.0083 |
| ***S25*** | 0.0031 | 0.0117 | 0.0168 | 0.0112 | 0.0214 | 0.0069 | 0.0048 | 0.0319 | 0.0082 | 0.0282 | 0.0503 | 0.0233 |
| ***MEAN*** | 0.0217 | 0.0162 | 0.0183 | 0.0220 | 0.0498 | 0.0696 | 0.0551 | 0.0675 | 0.0268 | 0.0170 | 0.0258 | 0.0204 |
| ***SD*** | 0.0179 | 0.0112 | 0.0139 | 0.0207 | 0.0414 | 0.0530 | 0.0414 | 0.0510 | 0.0203 | 0.0201 | 0.0180 | 0.0119 |

*Abbreviations*: lSTG = left STG, rSTG = right STG, lIFG = left IFG, rIFG = right IFG, V1 = Variation I, V2 = Variation II,

V3 = Variation III, V4 = Variation IV.
